# Detection and correction of probe-level artefacts on microarrays

**DOI:** 10.1186/1471-2105-13-114

**Published:** 2012-05-30

**Authors:** Tobias Petri, Evi Berchtold, Ralf Zimmer, Caroline C Friedel

**Affiliations:** 1 Institute for Informatics, Ludwig-Maximilians-Universität München, Munich 80333, Germany

**Keywords:** Microarrays, Quality control, Artefact detection

## Abstract

**Background:**

A recent large-scale analysis of Gene Expression Omnibus (GEO) data found frequent evidence for spatial defects in a substantial fraction of Affymetrix microarrays in the GEO. Nevertheless, in contrast to quality assessment, artefact detection is not widely used in standard gene expression analysis pipelines. Furthermore, although approaches have been proposed to detect diverse types of spatial noise on arrays, the correction of these artefacts is mostly left to either summarization methods or the corresponding arrays are completely discarded.

**Results:**

We show that state-of-the-art robust summarization procedures are vulnerable to artefacts on arrays and cannot appropriately correct for these. To address this problem, we present a simple approach to detect artefacts with high recall and precision, which we further improve by taking into account the spatial layout of arrays. Finally, we propose two correction methods for these artefacts that either substitute values of defective probes using probeset information or filter corrupted probes. We show that our approach can identify and correct defective probe measurements appropriately and outperforms existing tools.

**Conclusions:**

While summarization is insufficient to correct for defective probes, this problem can be addressed in a straightforward way by the methods we present for identification and correction of defective probes. As these methods output CEL files with corrected probe values that serve as input to standard normalization and summarization procedures, they can be easily integrated into existing microarray analysis pipelines as an additional pre-processing step. An R package is freely available from http://www.bio.ifi.lmu.de/artefact-correction.

## Background

Hybridization-based DNA microarrays are a key technology for high-throughput quantification of expression levels for thousands of genes [1,2]. State-of-the-art microarrays now allow the genome-wide analysis of transcript abundance not only for entire genes but also individual exons, alternatively spliced transcripts and even a large fraction of non-coding genomic regions [3,4]. Thus, despite the increasing prevalence of alternative methods such as RNA-seq [5], RNA microarrays remain important for the analysis of many biological processes such as miRNA-based regulation [6], alternative splicing patterns across human tissues [7] or the role of alternative splicing in stem cell differentiation [8] and cancer [9].

Recently, Langdon et al. [10] reported that *all* human Affymetrix microarrays available in the Gene Expression Omnibus (GEO) [11] contain spatial defects to some degree. Thus, quality control for microarrays remains a major issue. Although many methods and software tools have been developed for quality assessment of microarrays [12-15], detection of spatial artefacts is not yet routinely applied. Furthermore, it is usually not clear how to proceed once such artefacts have been detected. The two alternatives are (1) to either completely exclude or (2) to include the corresponding arrays for any subsequent analysis. In the first case, the corresponding measurements are not available for gene expression profiling and may even have to be repeated if they are crucial to the analysis. This can be cost-intensive, for instance if corresponding samples have been used up. In the second case, one has to assume that normalization and summarization methods can correct for the measurement errors.

The latter assumption is based on the construction of microarrays where probes of the same probeset are not contiguous on the array. Thus, smaller artefacts due to uneven hybridization or other experimental problems may only affect a subset of probes for a probeset. It is usually assumed that summarization methods – which combine the values for individual probes to a probeset value, such as *RMA*[16] – can estimate the probeset value correctly despite measurement errors for some probes.

In this study, we illustrate that this assumption is often invalid by showing that even small artefacts on the array can have a significant effect on the overall expression values of many probesets, not only the ones affected by the artefact. Furthermore, we introduce two simple but effective approaches for the identification of corrupted probes: (1) a threshold-based approach and (2) an extension of this approach that takes into account the neighborhood of a probe i.e. spatial information of the array. We show that the use of spatial information improves the identification of defective probes as well as reproducibility of probeset intensities after summarization. Finally, two strategies are proposed to either correct probe values using probeset information or filter corrupted probes, both of which improve summarization accuracy as well as reproducibility between replicates. In this way, we can recover even arrays with large artefacts for downstream analysis that otherwise would have to be discarded.

### Related work

Although not commonly included in standard microarray analysis pipelines, a number of methods have been previously proposed for visualization of microarray artefacts as well as identification and/or correction of corrupted probe measurements (see also [17] for an overview of methods published before 2008). One of the most frequently used approaches is *Harshlight*[18], which identifies and masks local artefacts based on statistical and image processing methods. Artefacts are grouped into three classes based on the variation from the median array: compact defects affecting only a few probes, diffuse defects affecting larger areas and extended defects that are even larger and may thus invalidate the whole array. Probes within defects can either be excluded from the analysis or be replaced by the median intensity across replicates.

An alternative method for identifying artefacts from raw intensity values is *Microarray blob remover (MBR)*[19], which operates in two steps. First, broad areas are determined in which more than half of the probes are above the *k*th percentile of probe intensities, where *k* may be in the range of 60 to 100. These candidate areas are then further refined and probes flagged to be within artefacts are added to the ‘outlier entries’ section in CEL files.

In addition, several other methods have been proposed based on comparisons to reference arrays [20-23]. Reimers and Weinstein [20] calculate the log fold-change of the probe value compared to the trimmed median of reference arrays to visualize spatial artefacts but do not aim to identify individual defective probes. Arteaga-Salas *et al.*[21] use a modification of the method by Upton and Lloyd [24] to identify areas in which the largest fold-changes compared to the median of all arrays all originate from the same array. Having identified arrays with defects, they then try to correct the original values using the values for the probe on the other arrays. A similar approach is also pursued by Hulsman *et al.*[22] as part of their normalization pipeline.

The most recent approach, *caCORRECT2*[23], uses a z-score-like statistic (*h*-score) to estimate whether a probe value on a given array is consistent with the observed distribution for all other arrays. Corrupted probes are then flagged if they have high *h*-scores and are contained in regions of high *h*-scores. Corrected values for these probes are then estimated both from the other probes in the same probeset as well as the other arrays using singular value decomposition.

## Results

### Outline and experimental setup

Our analysis is structured into two parts. First, we illustrate that state-of-the-art robust methods for summarization of probeset values from the individual probe values cannot appropriately correct for measurement artefacts. Second, we present methods for the identification of probes affected by measurement artefacts and the correction of artefacts by replacing the values of affected probes or modifying probeset definitions.

**Figure 1:**
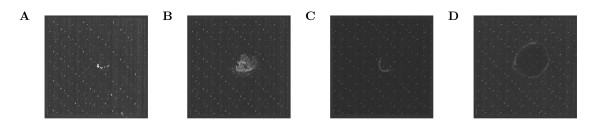
Measurement artefacts observed on different arrays of our dataset: total RNA for replicates 1 (a) and 3 (b) in DG75-10/12 cells; total RNA for replicate 2 (c) in DG75-eGFP cells; newly transcribed RNA for replicate 1 (d) in DG75-eGFP cells.

To evaluate the performance in correcting for artefacts, we used 18 exon array measurements of DG75 and DG75-10/12 B-cell lines (see Methods) for which distinctive measurement artefacts were observed in some samples (see Figure 1 and Additional file 1: Figure S1). These measurements included three replicates each of total RNA, newly transcribed RNA labeled for 60 min with 4-thiouridine [25,26] and the complementary unlabeled pre-existing RNA (see Methods for details). As newly transcribed and pre-existing RNA should sum up to total RNA, these experiments provide a true biological control for the assessment of quality problems and their correction (Figure 1).

In this study, we focused mostly on the measurements of the DG75-10/12 cells. In this case, 2 out of 3 total RNA measurements showed substantial spatial artefacts in the images of the arrays but the corresponding measurements of newly transcribed and pre-existing RNA were free of defects or showed only very small or weak artefacts allowing us to use these as biological control (see Additional file 1: Figure S1). The largest artefact affecting a sizable amount of probes was observed in replicate 3 and a smaller one in replicate 1. Replicate 2 was artefact-free in total RNA, although slight defects were observed in pre-existing and newly transcribed RNA. As we only required the total RNA sample of replicate 2 as a control for the other two replicates, this was not a problem.

### Identification of artefacts using probe noise plots

Although the array images already gave a first clue to the artefacts observed in our example, this was only due to the high intensity values of the affected probes and not all defects can be identified so easily. Thus, instead of intensities, we visualize *probe noise scores* that quantify the deviation from a control or a linear model as used e.g by *RMA*. As a control, we can use e.g additional replicates that are artefact-free in the relevant region or the biological control from RNA labeling experiments (see Methods for details). If residuals from the *RMA* models are used, the probe noise plots correspond to the *residual plot* proposed by Bolstad *et al.*[12].

Probe noise plots and residual plots for the arrays analyzed are shown in Additional file 1: Figure S2. Here, we used as controls for total RNA either the artefact-free replicate 2 or the normalized sum of newly transcribed and pre-existing RNA of the corresponding sample. While different noise scores pick up the artefacts similarly well for the defective replicates 1 and 3, a striking observation was made for replicate 2 in the *RMA* residual plots. Here, an additional stain showed up in the center of the array, which is not observed in the original image. Most likely, the *RMA* model, which is based on several replicates (in this case all three total RNA measurements), was biased by the stains on the other two arrays at this location, thus, leading to large residuals for replicate 2. This provides a first indication that summarization suffers from these artefacts.

### Insufficient correction by summarization

**Figure 2:**
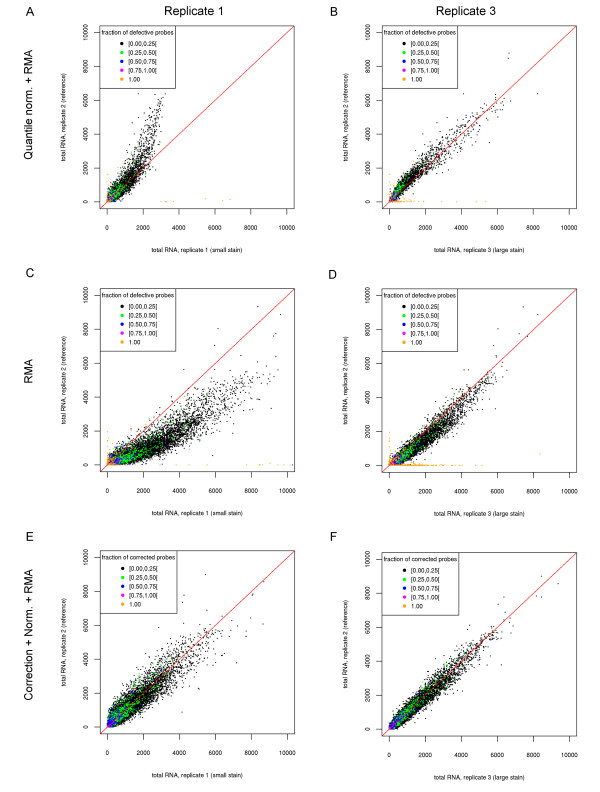
**Replicate scatter plots comparing total RNA for replicates 1 (a, c, e) and 3 (b, d, f) against the artefact-free replicate 2 for the exon array measurement in DG75-10/12 cells.** Subfigures **a** and **b** show the results using both *RMA* and quantile normalization, **c** and **d** using only *RMA* without quantile normalization and E and F after probe correction. Probesets are colored according to the percentage of their probes that are flagged as corrupted according to the *ε*-*criterion* based on the noise scores calculated using newly transcribed and pre-existing RNA as control. For replicate 1 there is a bias even for the uncorrupted probesets **(a)** that can be reduced by omitting quantile normalization **(c)**. If probe correction is applied prior to normalization and summarization **(e,f)**, this bias is removed. Here, the results are shown for the correction method which replaces the probe value by the mean of the unaffected probes in the same probe set. In this case, the intensity of probesets for which all probes are corrupted are set to zero. Results for the filtering approach in which affected probes are removed from the probeset definition are very similar.

To evaluate whether the final probeset values can nevertheless be estimated correctly by summarization, we used *replicate scatter plots* that compare probeset levels between the affected array and a control (see Figure 2 a,b and Additional file 1: Figure S3). Here, the same controls as for the probe noise plots were used and probesets were colored according to the fraction of probes that were flagged as corrupted by our simple thresholding approach on the probe noise scores (the *ε*-*criterion*, see Methods) (Figure 2).

As expected, the deviation to the control is substantial for probesets with all probes affected as no reasonable estimation is possible. In contrast, if only 75% or less (0-3 probes for most probesets) probes were affected, we did not see a correlation between the number of defective probes and the deviation from the diagonal. Instead, all probeset levels were affected to some degree. Strikingly, the deviation for replicate 1 with the small stain was stronger than for replicate 3 with the largest stain. Furthermore, this deviation was most pronounced for probesets with high expression values, which were not even affected by the stain.

One possible explanation is that this is an effect of the normalization – in this case quantile normalization – that has to be performed before summarization. It might compensate for the extremely high values for some of the probes by reducing the levels of the remaining probes. When omitting quantile normalization, the strong deviation for highly expressed genes in replicate 1 is reduced (Figure 2 c,d). Nevertheless, even without normalization, the nonlinear behavior for both replicates in comparison to replicate 2 is still observed.

Exon arrays also offer the possibility to summarize probe values to meta-probesets that correspond to genes. As there are more probes per meta-probeset the effect of the artefacts should be smaller. Nevertheless, we still observed a systematic shift from the diagonal in the corresponding replicate scatter plot, although for replicate 3 the deviation was much smaller (Additional file 1: Figure S4). In contrast to probeset level summarization, however, omitting quantile normalization did not reduce this deviation.

### Sensitivity of summarization to noise

To systematically analyze the influence of measurement artefacts on summarization, we performed the following experiment using three replicates of total RNA measured with exon arrays for the DG75-eGFP cell lines. These measurements were basically artefact-free with only a very small stain in one replicate, which could be easily corrected using our *ε*-*criterion* (Additional file 1: Figure S5). Here, only 6380 probes (out of >5.5 million features on the array) were identified as corrupted and 6335 probesets had 1 corrupted probe, 21 had 2 and only one had 3. This is a much smaller number than observed for the substantial artefacts on the DG75-1012 arrays.

We then introduced artificial measurement artefacts into the corrected DG75-eGFP arrays (*spiking*, see Methods). Depending on a noise level *δ*, probes to be spiked were chosen randomly with probability *δ*and their intensity values were drawn randomly from a log-normal distribution (with parameters $\mu =\underset{2}{\log}(850)$ and *σ*=1). Mean intensity values were taken from corrupted probes identified by the *ε*-*criterion* on the DG75-10/12 total RNA measurements (mean intensity values ∼850) to provide a realistic level of noise. Spiking was performed for only one of the arrays and the remaining arrays were used as control. After spiking the raw values on the array, we performed summarization and normalization. To assess the effect on the resulting probeset levels, we evaluated the average log_2_ fold-changes in probeset levels between each pair of spiked array and unspiked control for noise levels in the range of 0.01 to 0.1. For each noise level, random spiking was repeated 100 times.

**Figure 3:**
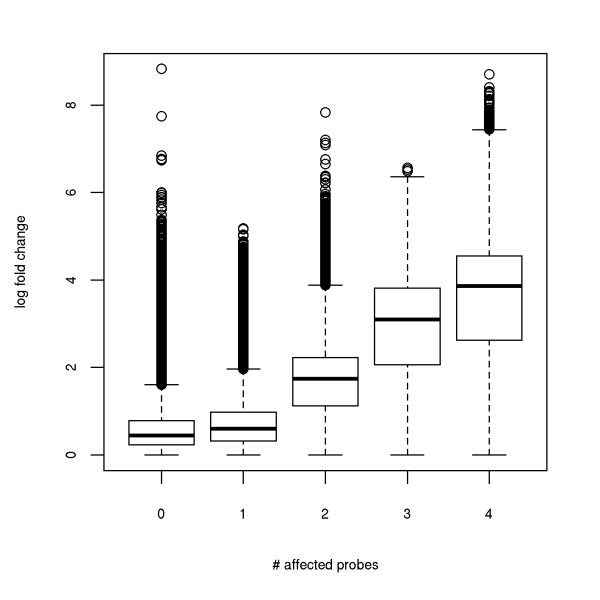
**Boxplot of the log_2_ fold changes for probesets with 0, 1, 2, 3 or 4 spiked probes in the simulation in which 5% of all probes were spiked in total (*δ*=0.05).** Here, probesets with the same number of spiked probes were pooled across all simulation results. For the case of 0 spiked probes, probesets were selected randomly from the pooled set as there were too many probesets for loading into R. In this case, each probeset was selected with a probability of 0.01. We observe a very strong correlation between the number of affected probes and fold-change biases on probeset level, which may seriously harm downstream analyses.

Comparing the log_2_ fold-change against the number of spiked probes for each probeset (Figure 3), we found a very clear trend: if only one probe is affected, the median fold-change is slightly higher than for probesets not affected by spiking. However, if more than one probe is spiked, the fold-changes increase substantially. Thus, variance of the probeset levels are increased considerably even if only few probes are affected. This larger variance can lead to low or no statistical significance for differentially expressed genes and as a consequence reduce the sensitivity of gene expression profiling (Figure 3).

### Identification and correction of corrupted probes

To address the problem of measurement artefacts for summarization, we propose a two-step approach in which we first identify corrupted probes and then correct for these corrupted probes in one of two ways. The first correction method consists in replacing the probe value by the mean of the remaining unaffected probes for the given probeset. The second alternative consists in removing the probe from the analysis, for instance by re-defining probeset definitions to exclude the affected probes. As several analysis tools including the Affy Power Tools (APT) suite cannot handle missing values appropriately and even the *de facto* standard of present and absent flags is often ignored by downstream tools, the first method is more robust than the second.

To identify the corrupted probes we use a simple threshold criterion based on probe noise scores calculated either from fold-changes to a control or *RMA*-derived residuals. Here, we developed two approaches that calculate the probe noise score either for each probe alone (*ε*-*criterion*) or as a distance-weighted mean of the noise scores within a 2D-window around the probe (*ε*-*criterion*) (see Methods for details). The latter approach is based on the observation that measurement artefacts, e.g. due to uneven hybridization, usually affect several closely located probes and not only individual probes. Probes with a noise score above a certain threshold are then flagged as corrupted.

To correct the DG75-10/12 measurements and to evaluate the performance of correction appropriately, we pursued the following procedure to avoid overfitting. If we compared the corrected and summarized probeset values between replicates (Figure 2 e-f), detection of corrupted probes was based on the ratio of total RNA to the normalized sum of newly transcribed and pre-existing RNA and vice versa (Additional file 1: Figure S6).

These results show a significant improvement after probe value correction. Both with and without quantile normalization, the distinctive deviation for large expression values seen before in replicate 1 is no longer observed. Instead, for both defective replicates 1 and 3, variance is symmetrical on both sides of the diagonal. This was true both for the correction using mean values of unaffected probes of the same probeset (Figure 2 e-f, Additional file 1: Figure S6) as well as for the filtering approach in which the affected probes were removed from the probeset definition (not shown). Here, the mean absolute deviation from the diagonal decreased from 12.2 in the original data to 7.34 and 4.7 for the first and second correction method, respectively. Thus, even the simple *ε*-*criterion* could successfully identify the defective probes and probeset values could be corrected appropriately, with slightly better results obtained by removing affected probes instead of using values from unaffected probes.

### Accuracy of artefact identification approaches

**Figure 4:**
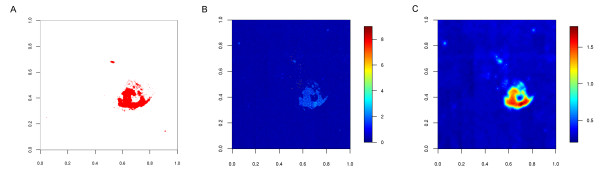
**Illustration of the results on the spiked Gene ST arrays.** Both shape of the artefact and intensities of the spiked probes were transfered from exon arrays containing artefacts. **a**) shows the spiked probes in red and **b**) and **c**) the probe scores based on fold changes between replicates using only the probe information itself (**b**) or also its neighborhood (**c**). For both **b** and **c** the overall shape of the spiked stain can easily be identified, but only when using the *window-criterion* (**c**) all probes within this area are identified. Furthermore, in B there are more probes with high noise scores that were not spiked (false positives).

To perform a systematic analysis of the performance in detecting measurement artefacts, we used Gene ST array measurements of the same samples that were measured with the exon arrays. The Gene ST measurements were free of artefacts and have been published recently [27]. Artifical stains were spiked into these artefact-free Gene ST measurements by projecting the artefact observed in total RNA of replicate 3 for the DG75-10/12 cells from the exon arrays to one sample of total RNA measured with gene arrays as described in the Methods section (Figure 4 a). We used the pattern of the stain on a real-life example instead of random selection or some other spatial pattern to perform a realistic simulation of noise and fair comparison of the approaches(Figure 4).

#### Compared methods

We compared the *ε*-*criterion* and *window-criterion* using probe noise scores based on 

1. fold-changes between replicates, calculated from all 3 replicates of total RNA for the DG75-eGFP cells including the spiked replicate.

2. fold-changes between total RNA and normalized sum of newly transcribed and pre-existing RNA corresponding to the spiked replicate.

3. *RMA* residuals calculated based on all 18 replicates using the *affyPLM* library.

These approaches were additionally compared against *Harshlight*[18] and *MBR*[19], which were applied to the 6 array measurements of total RNA. *Harshlight* does not provide noise scores per se but relies on downstream algorithms to decide on affected probes. To compute precision and recall values, probes were ranked by their fold-change to the corresponding median probe value across all arrays. This score is used by *Harshlight* in its initial step. For our purposes, it was additionally incremented by a constant value for probes flagged as defects by *Harshlight* such that all flagged probes ranked higher than any other probe. As *MBR* is only available as a GUI, we investigated only a small number of values for the parameter *k* (60-80 in increments of 5; values larger than 80 were found to have only very small recall).

Additionally, we planned to evaluate performance of *caCORRECT2*[23] as well as the method by Reimers and Weinstein [20], which both are available as webservers. However, as none of the two programs had yielded a result 24 h after uploading the data to the webservers, we aborted the evaluation. Thus, it appears that these methods did not scale well to the size of the Gene ST arrays used in this study, which are substantially larger than older Affymetrix arrays but still much smaller than the exon arrays. Alternatively, in particular for the Reimers and Weinstein method, the webservers might no longer be maintained. The method by Hulsman *et al.* for identifying location artefacts is only available as an intermediate step within their normalization pipeline and could not be evaluated on its own.

#### Evaluation results

Figure 4 illustrates the spiked artefact as well as the probe noise scores calculated using either only the probe information alone or including also the probe neighborhood using the window-based approach. Here, the probe scores were calculated from the fold-changes between replicates. The window approach results in a much smoother change of scores and high noise scores within the complete spiked area. If scores are calculated on each probe alone, we observe large variations in the spiked area with not all probes having high scores. Similar results are observed for the other types of noise scores (Additional file 1: Figure S7), indicating that the window approach results in higher sensitivity in identifying defective probes.

**Figure 5:**
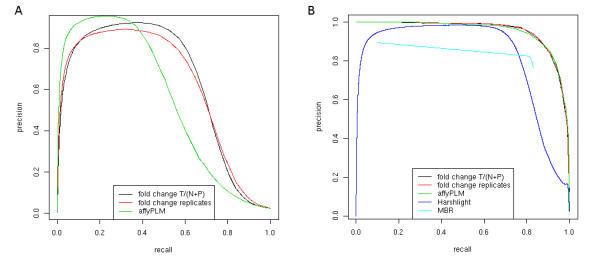
**Precision-Recall curves for spiked Gene ST measurements.** Here, artefacts were projected from the exon array measurements onto the gene arrays to produce realistic noise patterns. Three different scoring approaches were compared both for the simple threshold approach, the *ε*-*criterion* (**a**), and its cumulative variant, the *window-criterion* (**b**), which takes into account the probe neighborhood information. The scoring approaches compared are: (i) absolute log fold change between total RNA and normalized sum of newly transcribed and pre-existing RNA (*fold change (T/N + P)*, see Methods); (ii) absolute log fold change between replicates (*fold change replicates*); (iii) residuals determined with the *RMA* summarization approach using the *affyPLM* model (*affyPLM*). These results show that the window-based approach improves the performance of all used methods, resulting in almost identical performance for all of them, which is superior to the performance of both *Harshlight* and *MBR.*

To compare the different approaches, Precision-Recall curves were calculated (Figure 5). For this purpose, precision in identifying defective probes is plotted on the y-axis against recall on the x-axis for decreasing thresholds for flagging a probe corrupted. Here, several interesting observations can be made. First, the noise scores based on fold-changes to either replicate or newly transcribed plus pre-existing RNA samples perform almost identically using the *ε*-*criterion*. In contrast, the scores based on the *RMA* residuals show a higher precision for low recall values but this precision deteriorates more rapidly for increasing recall values (Figure 5).

Second, performance of all probe scores improves considerably when the *window-criterion* is applied. By taking the local information of a probe’s neighborhood into account, recall can be increased significantly while the number of probes mistakenly flagged as corrupted is reduced. Furthermore, when using the *window-criterion* the differences between the scoring approaches disappear and all scoring methods show a very similar performance. Here, the reason for the poor performance of the *ε*-*criterion* at low recall are a few isolated probes with high noise scores on the arrays that were not spiked and, thus, are counted as false positives. While these outliers might also be interesting, they do not indicate a systematic artefact. Accordingly, smoothing over the scores in the neighborhood of these probes reduces their noise level. This enables us to find an appropriate threshold between spiked and unspiked probes independent of the scoring method used.

Finally, the performance of the different *window-criterion* variants was compared to *Harshlight* and *MBR*. While *Harshlight* performs similarly well for intermediate recall values, precision is very low when trying to reach full recall. At a recall of 85% of spiked probes, the fraction of correctly flagged probes is only less than 50%, whereas for the *window-criterion* more than 90% of the flagged probes had been spiked. Thus, it appears that *Harshlight* uses too strict requirements on probe quality and, accordingly, tends to flag too many probes as defective. Additionally, modern platforms like gene and exon arrays appear to cause problems to *Harshlight* due to either calibration or technical issues. Using default settings large diffuse defects are detected even for artefact-free arrays and spike-in probes used for calibration are detected as compact defects.

*MBR* also performed worse than all *window-criterion* variants at all recall values but outperformed *Harshlight* in a small range. It should be noted that the parameter *k* used by *MBR* allowed only very little tuning of performance. For *k*=80 (the largest value investigated), recall was as low as 0.1, then increased dramatically to 0.81 for *k*=75 and then only increased moderately up to 0.83 for the smallest allowed value of *k*=60. At the same time, precision varied only between 0.90 for *k*=80 and 0.77 for *k*=60.

## Discussion

A recent study [10] showed that an alarming number of arrays deposited in the Gene Expression Omnibus (GEO) contain spatial defects. As deposition in the GEO generally occurs only prior to publication, this does not even include the array measurements that were discarded before analysis due to larger artefacts. If artefacts are not considered substantial enough to discard the array, normalization and summarization procedures are routinely relied upon to correct for these smaller defects.

In this study, we show that this reliance is risky as even small artefacts can have serious impacts on the results of normalization and summarization procedures. For this purpose, we used a set of exon array measurements which contained several artefacts of various sizes in the form of stains in the center of the arrays. In this case, replacement of the corresponding arrays by measurements of the same samples was not an option as for some samples all RNA material had been used up. Thus, the only alternatives were to either repeat the whole experiments and all array measurements which would be highly cost-intensive or try to use at least the measurements of the probes not affected by the artefacts.

However, the comparison of replicates containing artefacts with corresponding artefact-free measurements clearly showed that even a robust summarization procedure such as *RMA* could not appropriately correct for the defective probes as considerable non-linear deviations were observed between replicates. Interestingly, the degree of the deviations between replicates did not necessarily depend on the number of probes affected in total on the array. One of the arrays showed only a minor stain which would normally be considered acceptable. Yet, deviations compared to the control were substantial, in particular for high expression values. Although this effect could be traced partially to quantile normalization, it did not disappear when omitting normalization.

While simulations showed a clear correlation between the number of probes affected within a probeset and the deviation between replicates of the estimated probeset values, this was not quite reflected in the real-life measurements. Here, probesets were similarly affected no matter whether 1, 2 or 3 probes were corrupted. Only probesets for which all probes were defective were clear outliers. Furthermore, even probeset measurements with no corrupted probes were biased. To some degree this is due to the normalization step required before summarization, which is based on the global intensity distribution on the arrays. As even small artefacts on the arrays can have a substantial influence on the distribution of intensities, all normalized probe values are affected to some degree. Although it is not clear why this is effect is also seen without normalization, it can clearly be attributed to the artefacts as it disappears after correction of corrupted probes.

Although a few approaches have been proposed for identifying probe-level artefacts, they are usually highly complex using e.g. image analysis as in the case of *Harshlight* or singular value decomposition in case of *caCORRECT2*. Furthermore, they were often developed for previous generations of Affymetrix arrays and do not scale well to the new Gene and Exon ST array designs. Here, we propose a much simpler approach that relies only on the availability of an artefact-free control measurement, which is usually available in the form of technical or biological replicates. Instead of replicates, a biological control can alternatively be used as in the case of simultaneous measurements of total, newly transcribed and pre-existing RNA.

We showed that even a simple comparison of fold-changes between corrupted and control measurements was sufficient to identify the corrupted probes on our exon array measurements and correct them by either replacing their values or removing them from the analysis. While noise scores calculated only from the probe values themselves were already successful for this purpose, an extension of this approach that also uses the neighborhood of the probe improved the performance even more. In this case, the probe noise score is calculated as a distance-weighted average of all noise scores in a window around the probe considered. In this way, the layout of the array and the spatial nature of most artefacts can be taken into account. This approach was also superior to *MBR* as well as *Harshlight*, which in general appeared to be too stringent on probe quality as it also flagged many probes on arrays without apparent defects.

## Conclusions

In this article, we illustrate the necessity of integrating artefact detection and correction into standard gene expression analysis pipelines as state-of-the-art normalization and summarization procedures were found to be vulnerable even to small spatial defects. We propose a general and simple approach for identification and correction of these artefacts that relies only on the availability of controls in the form of technical or biological replicates. By additionally taking the probe neighborhood into account, we can furthermore improve detection accuracy also compared to more complex approaches. Thus, even if a substantial amount of probes is defective on an array, the measurements of the remaining probes can be still be used.

## Methods

### Microarray measurements

We used RNA measurements for two cell lines using both Affymetrix GeneChip Human Gene 1.0 ST and Exon 1.0 ST arrays: 1) the B-cell line DG75 transduced to express 10 out of 12 miRNAs encoded by the Kaposi’s sarcoma-associated herpesvirus (KSHV) (DG75-10/12) and 2) DG75 transduced to express eGFP (DG75-eGFP) as control [28]. For each cell line and array type, total RNA was quantified. In addition, RNA synthesis and decay was measured using a recently developed method for labeling of newly transcribed RNA using 4-thiouridine (4sU) [25,26]. This allows the separation of total RNA (*T*) into the labeled newly transcribed RNA (*N*) and the unlabeled pre-existing RNA (*P*) as well as quantification of de novo transcription and decay in a single experimental setting.

For each cell line and each RNA fraction three replicates were measured resulting in a total of 18 arrays for each microarray platform. The Gene 1.0 ST measurements were recently published [27]. Exon 1.0 ST measurements were performed in the same way. However, in this case considerable experimental artefacts were observed for several of the 18 arrays resulting in distinctive stains visible in the array images (see Figure 1 and Additional file 1: Figure S1). These artefacts were probably a consequence of a drying out of the central part of the array during the hybridization step resulting in artificially high values for the corresponding probes.

### Summarization and normalization

Two steps that are generally performed first in a microarray experiment are normalization and summarization. Normalization is applied to allow the comparison of results from different replicates and conditions. Summarization estimates overall expression values for each probeset from the individual probe measurements.

#### Normalization

In this study, we used quantile normalization, which is commonly used in combination with *RMA* summarization. If newly transcribed (*N*) as well as pre-existing (*P*) RNA have been quantified in addition to total cellular RNA (*T*), an additional normalization step has to be applied to account for the different amounts of RNA between the fractions [26]. Since *T*=*N* + *P* has to hold approximately for all probes, the linear model *T*=_*λ*1_*N* + _*λ*2_*P*minimizes the sum of residuals for $\lambda_{i}\in R^{+},\;i\in \{1,2\}$. The corresponding _*λi*_can be found by linear regression [26], which can be applied both on the summarized probeset values as well as the individual probe values themselves. If the fold-change between replicates is used to calculate the probe noise score a loess normalization is additionally applied before fold-change calculation.

#### Summarization

One of the most widely used summarization methods is *RMA*[16], which estimates both an overall expression value for each probeset and the probe-specific measurement error by fitting a linear model to the probe values. Thus, this method implicitly estimates the noise level for each probe and effectively subtracts the estimated noise from the probe when calculating the overall probeset values. This explains why it is commonly assumed that this method can correct for measurement artefacts [29]. For our purposes, the Affymetrix Power Tools (APT) were used for summarization ( http://www.affymetrix.com). An alternative implementation is provided by the *affyPLM*[12] library, which also provides access to the estimated residuals. However, due to its considerable memory usage when working on exon arrays the *affyPLM* library could not be applied to all exon array measurements together.

### Quality assessment

#### Probe noise score

To assess the level of noise for individual probes, different criteria can be applied. If measurement errors are explicitly modeled as in the *RMA* approach, residuals can be used to assess reliability of the corresponding approach [12]. The higher the absolute values of the residuals, the stronger the effect of measurement errors on this probe. The global residual level (e.g. calculated by the APT subroutine *qcc*, [29]) can be used to indicate which arrays are suited as control and which are likely to contain artefacts.

A general probe-level noise score for probe *j* can be calculated as the fold-change compared to a control:

(1) $$\begin{array}{ll} s_{j}=\left|\underset{2}{\log}\frac{v_{j}+c}{v_{j}^{'}+c}\right| & \; \end{array}$$

Here, *v*_*j*_ is the intensity for the probe on the corrupted array and *v*_*j*_^*'*^is their value on the control. The pseudocount *c* corresponds to the estimated detection limit (in our case *c*=16). Both *v*_*j*_ and *v*_*j*_^*'*^ can be measured directly or can be derived values, e.g. using measurements of newly transcribed and pre-existing RNA as described in the normalization section. In the latter case, the normalized sum of *N* and *P* serves as a control for the measurement of *T*, i.e. *v*_*j*_=*T*and *v*_*j*_^*'*^=_*λ*1_*N* + _*λ*2_*P*. Alternatively, replicates may serve as a control. If it is not possible to determine a suitable control, the fold-change against the median probe intensities of all replicates can be used. This corresponds to the error image used by *Harshlight*[18].

#### Probe noise plot

As each probe has a defined location on the array, the noise level of individual probes can be visualized by plotting the noise score of the probe against this location. For a more intuitive visualization, the noise score is color-coded and the location represented by the *x*- and *y*-axis. If residuals from the *RMA* model are plotted, this corresponds to the *residual plot* proposed by Bolstad et al. [12]. To calculate residuals for noise plots, we used the *affyPLM* implementation of *RMA* which provides access to these residuals. In this case, residual estimation for a specific array was based on the three replicates for the corresponding condition.

#### Replicate scatter plot

To evaluate correction of artefacts, probeset values for the affected arrays are plotted against the control values. If no artefacts are observed, summarized probeset values should be highly reproducible between the replicates. Instead of replicates for the same condition and RNA fraction, the complementarity of the total, newly transcribed and pre-existing RNA fractions can be exploited.

#### Introducing artificial noise

Measurement errors were introduced artificially (*spiked*) in exon array measurements by selecting a noise level *δ*and spiking each probe according to this probability. The raw measured values of spiked probes were then replaced by an artificial level drawn from a log-normal distribution with mean *μ* and standard deviation *σ* (in our case $\mu =\underset{2}{\log}(850)$ and *σ*=1 were inferred from the intensities within the real artefacts). Only probes corresponding to core probesets defined by Affymetrix were spiked and included in the summarization. Simulations for each selected value of *δ* were repeated 100 times.

Furthermore, real-life stains were projected onto the Gene ST arrays to create realistically shaped artefact patterns. For this purpose, our artefact detection approach (see below) was applied to the exon array measurements with artefacts to detect the location of the corrupted probes. The exon array artefacts were then scaled down to the dimensions of the gene arrays and transferred to the artefact-free gene arrays. For this purpose, 2×2 rectangles of probes on the exon arrays was mapped to one probe on the gene arrays and the maximum value of any of the probes in this rectangle was used for the spiked probe. To account for the overall larger intensities on the gene arrays, the resulting value was multiplied by the ratio of the 75 percentile of the intensity distribution on the gene arrays relative to the corresponding 75 percentile for the exon arrays.

### Artefact detection

We propose two alternative approaches to identify probes which are affected by significant measurement errors. The first method is based on a simple threshold criterion, the second approach extends this method by including the neighborhood information on the array.

#### *ε*-criterion

The *ε*-criterion is based on the noise score defined in equation 1 and simply applies a threshold *t* on this score. Thus,

(2) $$\varepsilon (s_{j})=\left\{\begin{array}{cc} \text{true} & \;s_{j}>t\\ \text{false} & \;\text{otherwise} \end{array}\right)$$

If *ε*(*s*_*j*_) is true, probe *j* is flagged as corrupted. Thresholds can be adjusted manually by analyzing both probe noise and replicate scatter plots.

#### Window criterion

As measurement artefacts usually affect a specific region on the array and, accordingly, a set of probes closely located to each other, we propose a method which takes into account the neighborhood information. Thus, for estimating the reliability of a specific probe we take into account the values of the probes in a window around this probe. For our purposes, we used a 2D window of dimension (2*k* + 1)×(2*k* + 1) with the probe considered in the center of the window (here *k*=25 was used). We calculate a weighted average of the probe noise scores in this window:

(3) $$sw_{j}=\frac{\underset{p\in P}{\sum }s_{p}\cdot w(p,j)}{\underset{p\in P}{\sum }w(p,j)}$$

where *P* is the set of probes in the window, _*sj*_the noise score of the probe *p* and *w*(*p*,*j*) is the weight of probe *p* in the window for *j*. The weight is calculated as 1/*d*(*p*,*j*) if *p*≠*j* where *d* is the distance between probes. In this study, we used the euclidean distance on the probe coordinates but alternative distances can be used. If *p*=*j*, the weight is set to 2. If residuals from *RMA*-like methods are used as noise scores, _*sp*_ is set to the absolute value of the residuals. For probes close to the borders of the array, the window will be cut off at the respective sides. Subsequently, the *ε*-*criterion* is applied to the window-based noise scores.

### Correction of corrupted probes

For correction of corrupted probe values we use two alternative approaches. In the first case, we replace the intensities of the corrupted probes by the mean intensity of the remaining probes of the corresponding probeset in the CEL file. This correction only takes into account probe values measured with the same array, thus, differences in intensity distributions between arrays do not have to be considered. If all probes of a probeset are corrupted it is not possible to infer a meaningful probeset intensity. Thus, we set all probe intensities to 0 resulting in a probeset intensity of 0. These probesets should be excluded from further analysis.

The alternative method consists in removing corrupted probes from the probeset definition by modifying the PGF annotation file provided by Affymetrix. It should be noted that it is also possible to completely exclude affected probes from the summarization procedure using the “--kill-list” option of the Affymetrix Power Tools, but it is still experimental and does not always work. Additionally, since downstream tools may request the filtered probe values direct probe value correction is far more robust than complete removal.

### Evaluation of artefact detection

To evaluate the performance of artefact detection, Gene ST arrays were spiked as described above. For each threshold applied, we then calculated *true positives* (spiked probes that are filtered, *TP*), *false positives* (probes not spiked but filtered, *FP*) as well as *true negatives* (probes neither spiked nor filtered, *TN*) and *false negatives* (spiked probes not filtered, *FN*). To evaluate different approaches over all possible thresholds, we used Precision-Recall curves for which

(4) precision=TP/(TP+FP)

is plotted on the y-axis against

(5) recall=TP/(TP+FN)

on the x-axis for all possible thresholds.

## Availability and requirements

• **Project name:** artefactCorrection

• **Project home page:**http://www.bio.ifi.lmu.de/artefact-correction

• **Operating system(s):** Platform independent

• **Programming language:** R

• **License:** GNU General Public License v3.0 (GPL-3)

• **Any restrictions to use by non-academics:** none

## Competing interests

The authors declare that they have no competing interests.

## Author’s contributions

TP, RZ and CCF designed the study and analyzed the results. EB implemented the methods and carried out the study. The manuscript was written by TP, EB and CCF. All authors read and approved the final manuscript.

## Supplementary Material

Additional File 1 Supplementary Figures. Click here for file
