# Supplementary material for: Detection and correction of probe-level artefacts on microarrays
Source: BMC Bioinformatics. 2012 May 30;13:114. doi: 10.1186/1471-2105-13-114 (PMC3534149; doi:10.1186/1471-2105-13-114)

Detection and correction of probe-level measurement artifacts

Tobias Petri, Evi Berchtold, Ralf Zimmer, Caroline C. Friedel

Supplementary Figures

Supplementary Figure 1

In the following, probe intensities are shown for the exon array measurements in DG75-eGFP and DG75-10/12 cells. Three replicates each were measured for newly transcribed and pre-existing RNA (see article) in addition to total RNA.

total RNA	newly transcribed RNA	pre-existing RNA
	DG75-eGFP replicate 1	
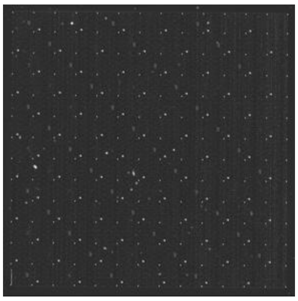	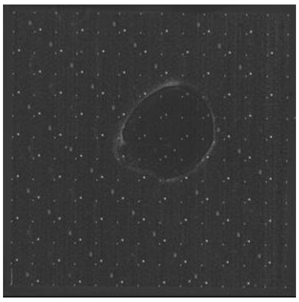	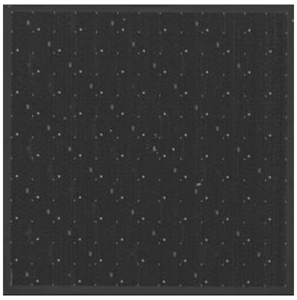
	DG75-eGFP replicate 2	
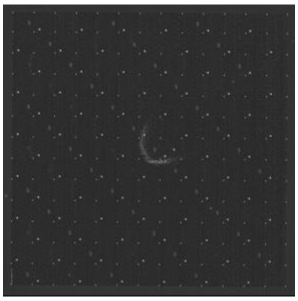	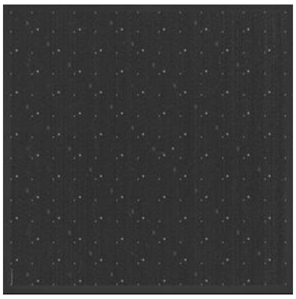	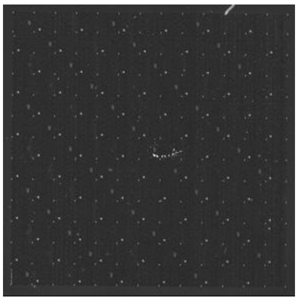
	continued on next page	

total RNA	newly Transcribed RNA	pre-existing RNA
	DG75-eGFP replicate 3	
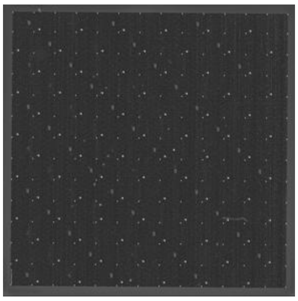	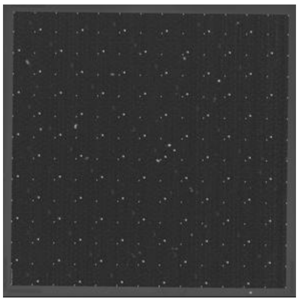	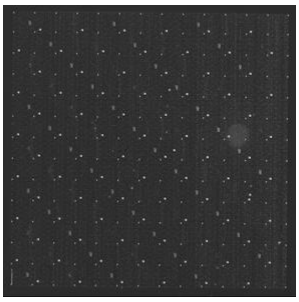
	DG75-10/12 replicate 1	
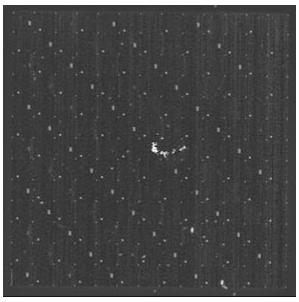	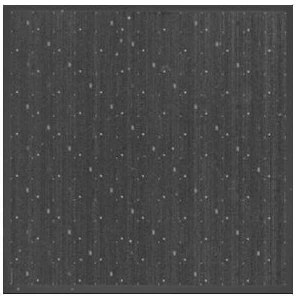	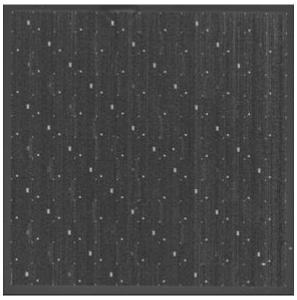
	DG75-10/12 replicate 2	
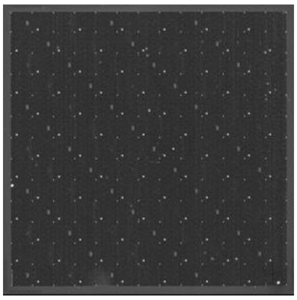	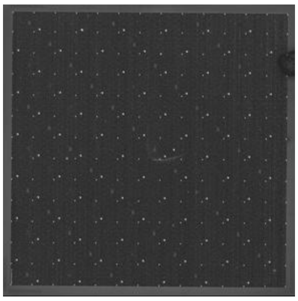	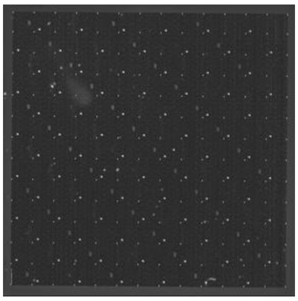
	DG75-10/12 replicate 3	
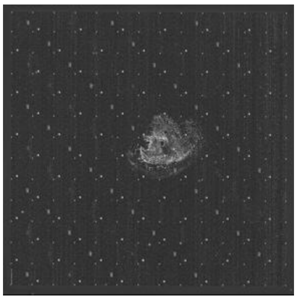	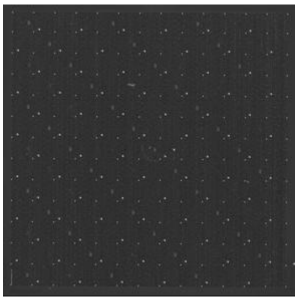	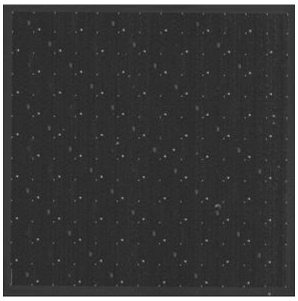

Supplementary Figure 2

Probe noise scores for the three replicates of total RNA in DG75-10/12 cells measured with exon arrays. From left to right

- original intensities,
- the noise score based on the fold-changes of total RNA to the sum of newly transcribed and pre-existing RNA (complementary score),
- the *affyPLM* residuals and
- noise scores based on the fold-change between replicates.

As replicate 2 was used as control in the latter case, no probe noise plot could be created based on replicate noise scores for this replicate.

Raw intensity	Complementarity score	*affyPLM* residuals	Replicate fold change

DG-75 10/12 replicate 1

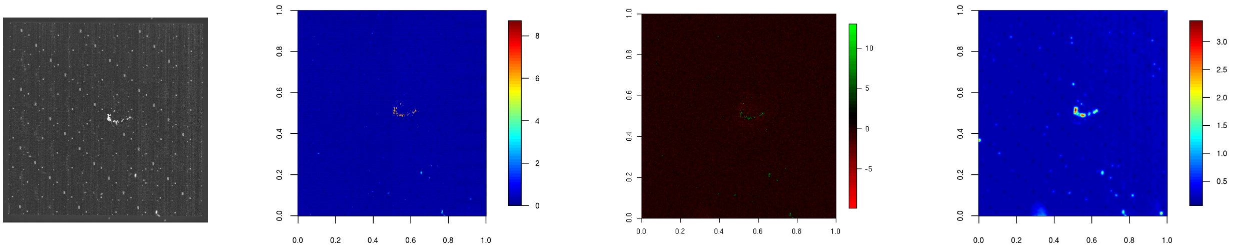

DG-75 10/12 replicate 2

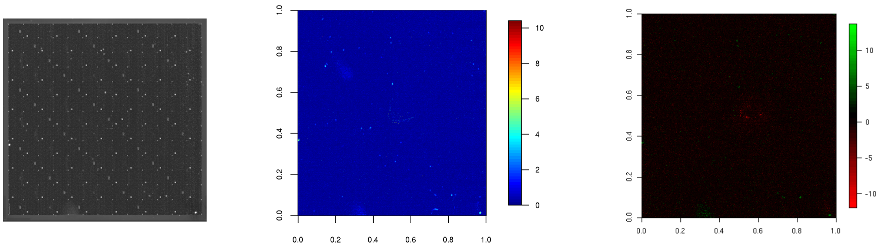

DG-75 10/12 replicate 3

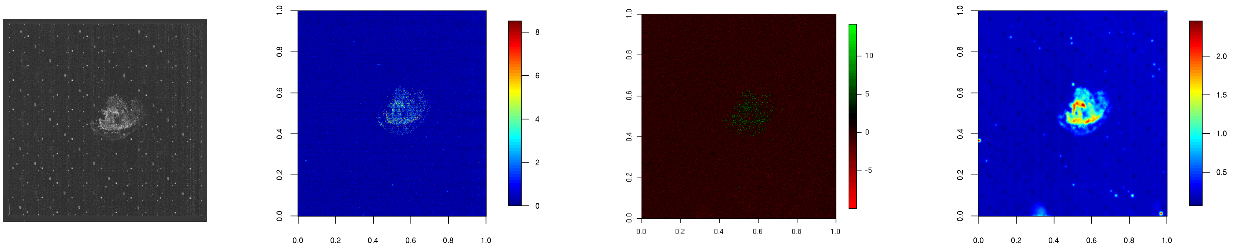

Supplementary Figure 3

Replicate scatter plots comparing total RNA against normalized sum of newly-transcribed and pre-existing RNA for replicates 1 (A, D), 2 (B, E) and 3 (C,F) for the DG75-10/12 exon array measurements. Subfigures A-C show the results using both RMA and quantile normalization, D-F using only RMA without quantile normalization. Here, the y-axis shows the control for the total RNA measurements. Deviations are observed for the two replicates 1 and 3 containing artefacts.

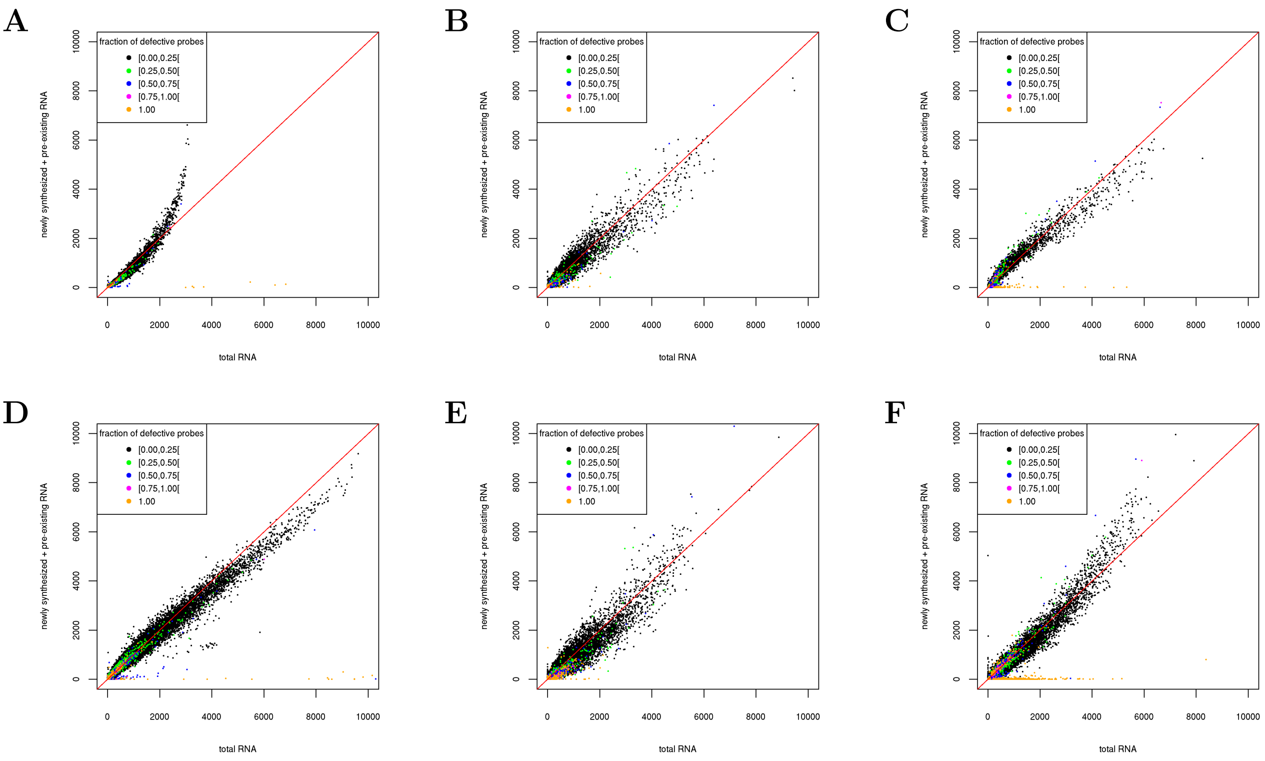

Supplementary Figure 4

Replicate scatter plots for the DG75-10/12 cells summarized to meta-probesets before (A,B) and after probe correction (C,D) with the ϵ-criterion using probe noise scores calculated based on the complementarity of RNA fractions.

replicate 1 replicate 3

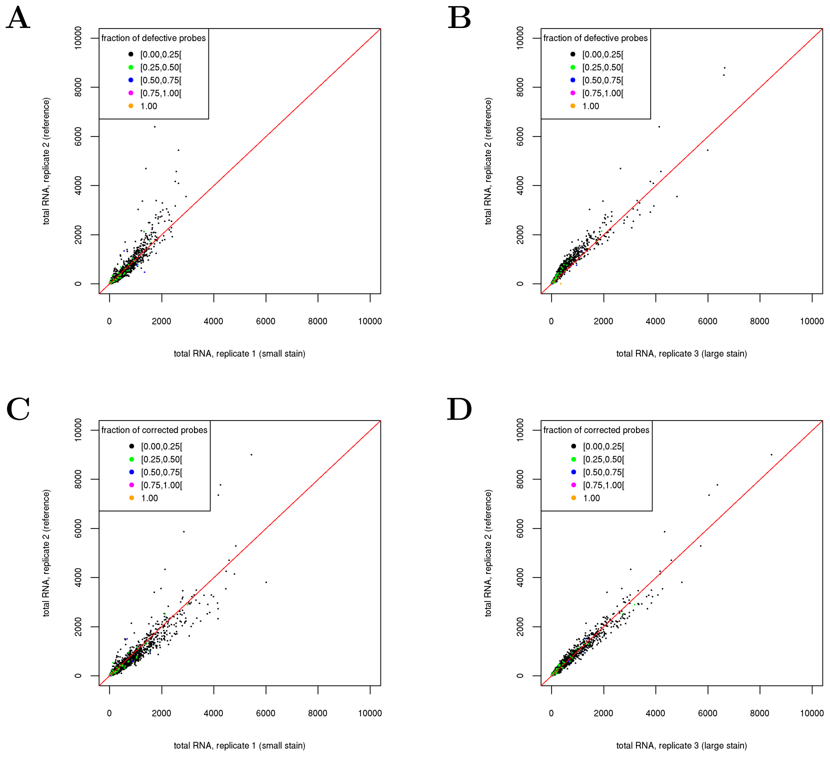

Supplementary Figure 5

Replicate scatter plots for the DG75-eGFP cells before (A-C) and after (D-F) probe correction with the ϵ-criterion with noise scores based on the complementarity of RNA fractions. Here, replicates 1 and 3 were free of artifacts and replicate 2 showed a small stain which results in deviations for this replicate. Here, replicate scatter plots for each pair of replicates are shown. Accordingly, deviations are observed in all replicate scatter plots for which replicate 2 has been used (A,C) but not in the comparison of the artefact-free replicates 1 and 3 (B). After probe correction no such deviation is observed even for replicate 2.

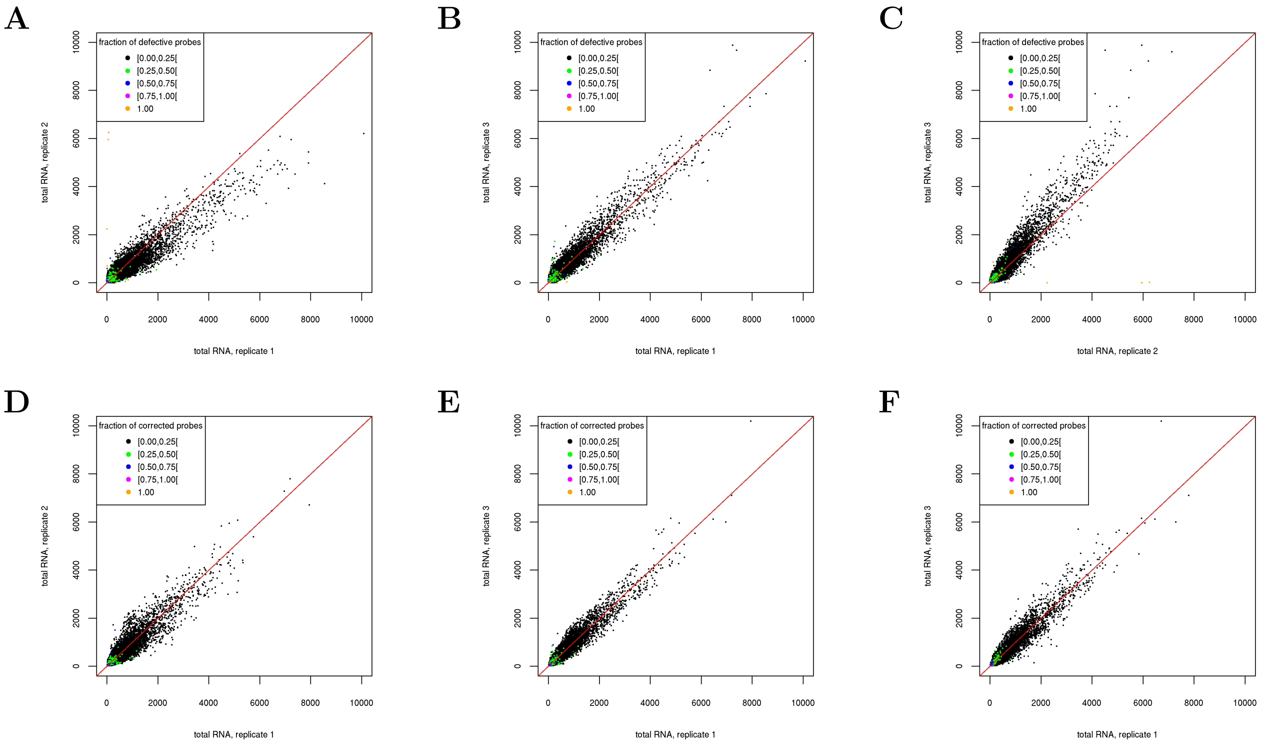

Supplementary Figure 6

Replicate scatter plots for the DG75-10/12 total RNA measurements after probe correction using the ϵ-criterion with probe noise scores based on replicate fold-changes (A: replicate 1, B: replicate 2 and C: replicate 3). To avoid overfitting effects, the total RNA is scattered against the normalized sum of newly-synthesized and pre-existing RNA.

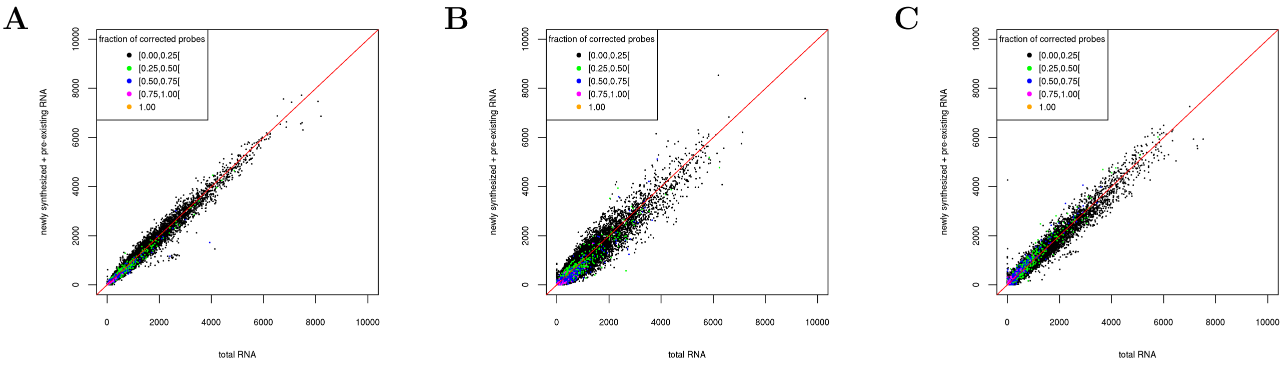

Supplementary Figure 7

Probe noise plots for the spiked Gene ST arrays using the the probe scores based on the comparison of replicates (A-B), the comparison of total RNA against the normalized sum of newly transcribed and pre-existing RNA (Complementarity score) (C-D) and the affyPLM residuals (E-F).

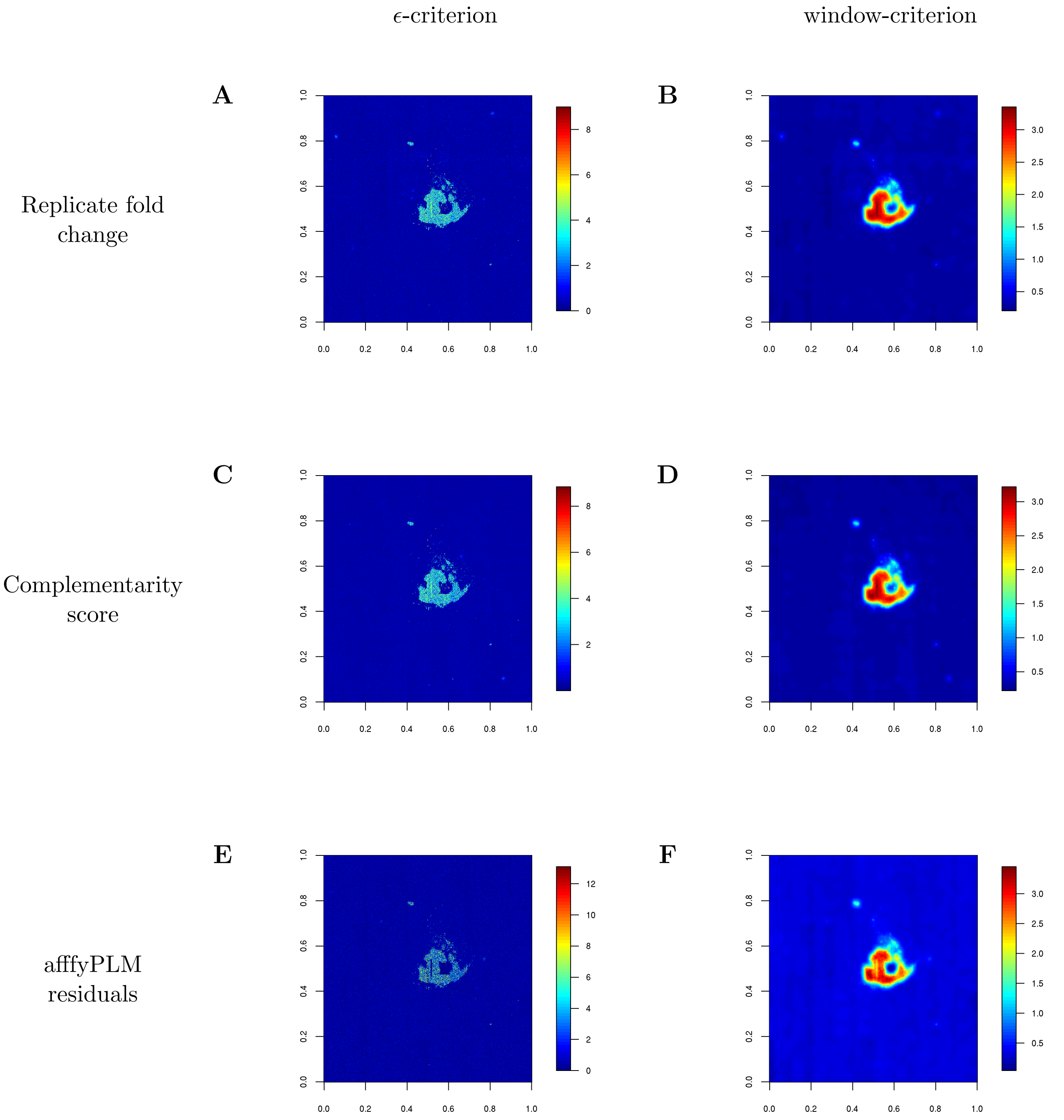

Supplement: Additional File 1 — Supplementary Figures. [file 1471-2105-13-114-S1.pdf]
